# Phosphodiesterases 3 and 4 Differentially Regulate the Funny Current, I_f_, in Mouse Sinoatrial Node Myocytes

**DOI:** 10.3390/jcdd4030010

**Published:** 2017-08-01

**Authors:** Joshua R. St. Clair, Eric D. Larson, Emily J. Sharpe, Zhandi Liao, Catherine Proenza

**Affiliations:** 1 Department of Physiology and Biophysics, University of Colorado School of Medicine, Aurora, CO 80045, USA; joshua.stclair@ucdenver.edu (J.R.S.); eric.larson@ucdenver.edu (E.D.L.); emily.sharpe@ucdenver.edu (E.J.S.); zdliao@ucdavis.edu (Z.L.); 2 Department of Medicine, Division of Cardiology, University of Colorado School of Medicine, Aurora, CO 80045, USA

**Keywords:** sinoatrial node, HCN channel, funny current (I_f_), cardiac pacemaking, cardiomyocyte, cell compartmentalization, phosphodiesterases, cyclic AMP (cAMP), ion channel, patch clamp

## Abstract

Cardiac pacemaking, at rest and during the sympathetic fight-or-flight response, depends on cAMP (3′,5′-cyclic adenosine monophosphate) signaling in sinoatrial node myocytes (SAMs). The cardiac “funny current” (I_f_) is among the cAMP-sensitive effectors that drive pacemaking in SAMs. I_f_ is produced by hyperpolarization-activated, cyclic nucleotide-sensitive (HCN) channels. Voltage-dependent gating of HCN channels is potentiated by cAMP, which acts either by binding directly to the channels or by activating the cAMP-dependent protein kinase (PKA), which phosphorylates them. PKA activity is required for signaling between β adrenergic receptors (βARs) and HCN channels in SAMs but the mechanism that constrains cAMP signaling to a PKA-dependent pathway is unknown. Phosphodiesterases (PDEs) hydrolyze cAMP and form cAMP signaling domains in other types of cardiomyocytes. Here we examine the role of PDEs in regulation of I_f_ in SAMs. I_f_ was recorded in whole-cell voltage-clamp experiments from acutely-isolated mouse SAMs in the absence or presence of PDE and PKA inhibitors, and before and after βAR stimulation. General PDE inhibition caused a PKA-independent depolarizing shift in the midpoint activation voltage (V_1/2_) of I_f_ at rest and removed the requirement for PKA in βAR-to-HCN signaling. PDE4 inhibition produced a similar PKA-independent depolarizing shift in the V_1/2_ of I_f_ at rest, but did not remove the requirement for PKA in βAR-to-HCN signaling. PDE3 inhibition produced PKA-dependent changes in I_f_ both at rest and in response to βAR stimulation. Our results suggest that PDE3 and PDE4 isoforms create distinct cAMP signaling domains that differentially constrain access of cAMP to HCN channels and establish the requirement for PKA in signaling between βARs and HCN channels in SAMs.

## 1. Introduction

Cardiac pacemaking, at rest and during the sympathetic fight-or-flight response, depends on cAMP signaling in sinoatrial node myocytes (SAMs). SAMs are highly-specialized cells that drive pacemaking by firing spontaneous action potentials (APs). Spontaneous APs in SAMs result from a spontaneous depolarization during diastole that drives the membrane potential to its threshold to initiate the subsequent AP. The diastolic depolarization in SAMs arises as a function of the coordinated activity of a unique complement of ion channels that work in concert with intracellular Ca^2+^ signaling [1,2,3,4]. 3′,5′-cyclic adenosine monophosphate (cAMP) is a critical regulator of pacemaking in SAMs. The resting cytoplasmic concentration of cAMP is thought to be higher in SAMs than in other cardiac myocytes [5] and sympathetic nervous system stimulation increases heart rate by activating β adrenergic receptors (βARs) and further increasing cAMP in SAMs.

The “funny current” (I_f_) is a hallmark of SAMs and is among the many cAMP-sensitive effectors that contribute to spontaneous pacemaker activity in SAMs. I_f_ is produced by hyperpolarization-activated, cyclic nucleotide-sensitive (HCN) ion channels. HCN4 is the predominant HCN channel isoform in the sinoatrial node of all mammals; it is expressed at high levels in SAMs and is used as a marker of the sinoatrial node [6,7,8,9]. I_f_ is activated by membrane hyperpolarization and is a mixed cationic conductance with a reversal potential of approximately −30 mV in physiological solutions [10,11]. Thus, I_f_ is inward at diastolic potentials, and it is thought to contribute to the diastolic depolarization phase of the sinoatrial AP. In accordance with a critical role for I_f_ in pacemaking, mutations in HCN4 channels cause sinoatrial node dysfunction in human patients and animal models [12,13,14] and HCN channel blockers decrease the heart rate [15,16].

cAMP potentiates voltage-dependent gating of HCN4 channels either by binding directly to a conserved cyclic nucleotide binding domain in the proximal C-terminus [17,18] or by protein kinase A (PKA)-mediated phosphorylation of the distal C-terminus [11]. In either case, cAMP causes a depolarizing shift in the midpoint activation voltage (V_1/2_). We previously showed that PKA activity is necessary for cAMP-dependent signaling between βARs and HCN channels in SAMs; inhibition of PKA with an inhibitory peptide, PKI, significantly reduced the shift in V_1/2_ produced by βAR stimulation [11]. However, we have also shown that HCN channels in SAMs can be activated by cAMP even in the absence of PKA activity [19], presumably by binding directly to the channels. Thus, the requirement for PKA in βAR-to-HCN4 channel signaling in SAMs could arise as a function of compartmentalization or restricted diffusion of cAMP [19].

Although cAMP is a small, soluble molecule, it does not behave as a freely-diffusing molecule in many types of cells [20,21,22,23,24,25,26,27]. cAMP concentration in cells is determined by a balance between production by adenylyl cyclases and degradation by cyclic nucleotide phosphodiesterases (PDEs) PDEs have been shown to form functional diffusion barriers in other types of cardiac myocytes [28,29,30,31,32]. PDEs are organized into 11 families, of which the PDE3 and PDE4 families are the most abundant in the mouse sinoatrial node [33]. Functional studies using subtype-specific inhibitors have shown that the PDE3 and PDE4 families regulate the beating rate of mouse right atrial preparations [34], as well as the AP firing rate and Ca^2+^ currents in isolated mouse SAMs [33]. PDE4 is specific for cAMP, although it has a relatively low affinity (2–8 µM). In contrast, PDE3 can hydrolyze both cAMP and cGMP, but has a high affinity for cAMP (10–100 nM). Hydrolysis of cAMP by PDE3 is inhibited by cGMP due to a ~10-fold slower maximum reaction rate for cGMP [35,36].

In this study we tested the hypothesis that PDEs contribute to regulation of I_f_ in SAMs by creating functional cAMP signaling domains. Indeed, we found that the PDE3 and PDE4 isoforms play distinct roles in regulation of I_f_, such that PDE4s control access of cAMP to HCN channels at rest, while PDE3s interact functionally with PKA to constrain signaling between βARs and HCN channels in SAMs.

## 2. Materials and Methods

### 2.1. Ethical Approval

This study was carried out in accordance with the US Animal Welfare Act and the National Research Council’s *Guide for the Care and Use of Laboratory Animals* and was conducted according to a protocol that was approved by the University of Colorado-Anschutz Medical Campus Institutional Animal Care and Use Committee (protocol number 84814(06)1E). Six- to eight-week old male C57BL/6J mice were obtained from Jackson Laboratories (Bar Harbor, ME, USA; Cat. #000664). Animals were anesthetized by isofluorane inhalation and euthanized under anesthesia by cervical dislocation.

### 2.2. Sinoatrial Myocyte Isolation

Sinoatrial myocytes were isolated as we have previously described [11,19,37,38,39,40,41,42]. Briefly, hearts were removed into heparinized (10 U/mL) Tyrodes solution at 35 °C (in mM: 140 NaCl, 5.4 KCl, 1.2 KH_2_PO_4_, 1.8 MgCl_2_, 1 CaCl_2_, 5 HEPES, and 5.55 glucose, with pH adjusted to 7.4 with NaOH). The sinoatrial node, as defined by the borders of the crista terminalis, the interatrial septum, and the inferior and superior vena cavae, was excised and digested in an enzyme cocktail consisting of collagenase type II (Worthington Biochemical, NJ, USA), protease type XIV (Sigma Aldrich, St. Louis, MO, USA), and elastase (Worthington Biochemical, Lakewood, NJ, USA) for 25–30 min at 35 °C in a modified Tyrodes solution (in mM: 140 NaCl, 5.4 KCl, 1.2 KH_2_PO_4_, 5 HEPES, 18.5 glucose, 0.066 CaCl_2_, 50 taurine, and 1 mg/mL BSA; pH adjusted to 6.9 with NaOH). Tissue was transferred to a modified KB solution (in mM: 100 potassium glutamate, 10 potassium aspartate, 25 KCl, 10 KH_2_PO_4_, 2 MgSO_4_, 20 taurine, 5 creatine, 0.5 EGTA, 20 glucose, 5 HEPES, and 0.1% BSA; pH adjusted to 7.2 with KOH) at 35 °C, and cells were dissociated by trituration with a fire-polished glass pipet for ~10 min. Ca^2+^ was gradually reintroduced, and dissociated cells were maintained at room temperature for up to 8 h prior to electrophysiological recordings.

### 2.3. Sinoatrial Myocyte Electrophysiology

For electrophysiology, an aliquot of the sinoatrial node myocyte suspension was transferred to a glass-bottomed recording chamber on the stage of an inverted microscope. Individual SAMs were identified by spontaneous contractions, characteristic morphology [11,19,37,38,39,40,41,42], capacitance <45 pS, and the presence of I_f_. Borosilicate glass pipettes had resistances of 1–3 MΩ when filled with an intracellular solution containing (in mM): 135 potassium aspartate, 6.6 sodium phosphocreatine, 1 MgCl_2_, 1 CaCl_2_, 10 HEPES, 10 EGTA, 4 Mg-ATP; pH adjusted to 7.2 with KOH. SAMs were constantly perfused (1–2 mL/min) with Tyrodes solution containing 1 mM BaCl_2_ to block K^+^ currents. A 1 mM stock solution of isoproterenol hydrochloride (ISO; Calbiochem/EMD Millipore, Billerica, MA, USA) in 1 mM ascorbic acid was stored as frozen aliquots, which were thawed on the day of experimentation and added to the perfusing Tyrodes solution to a final concentration of 1 μM as indicated. 

Whole cell voltage clamp recordings were performed >2 min after achieving the whole cell recording configuration, to allow for intracellular perfusion with the pipette solution. To determine the voltage dependence of I_f_, families of currents were elicited by 3 s hyperpolarizing voltage steps ranging from −60 mV up to −170 mV in 10 mV increments from a holding potential of −35 mV, as previously described [11,19,37,38,39,40,41,42]. Although steady state activation of I_f_ is not attained within 3 s for more depolarized potentials owing to the very slow kinetics of activation of I_f_, the protocol is an experimentally-feasible means to approximate and compare the voltage-dependence of activation of I_f_ in the presence of different inhibitors (see [11]). Conductance (*G*) was calculated from the inward currents as:

*G* = *I*/(*V*_*m*_ − *V*_*r*_) (1)

where *I* is the time-dependent component of I_f_, *V_m_* is the applied membrane voltage (corrected for a +14 mV junction potential error, calculated using JPCalc [43]), and *V_r_* is the reversal potential for I_f_ under these experimental conditions (−30 mV; [10,11]). Conductances were plotted as a function of voltage, and isochronal midpoint activation voltages (V_1/2_) were determined for each cell by fitting with a Boltzmann function:

(2) $$f(V)=V_{\min}+\frac{V_{\max}-V_{\min}}{1+e^{\frac{z_{d}F}{RT}(V-V_{1/2})}}$$

where *V*_min_ and *V*_max_ are the voltages corresponding to the minimum and maximum currents, *Z_d_* is the charge valence, *R* is the gas constant, *T* is temperature, and *F* is the Faraday constant. The conductance-voltage relationship was determined for each individual cell included in the study and all individual GVs reached saturation. Averaged GV relationships are extrapolated to −170 for all conditions to facilitate comparisons. All experiments were conducted at room temperature in order to access the full range of activation midpoints for I_f_ (approximately −110 to −130 mV). Three cells were considered outliers and were excluded from the datasets because their midpoint activation voltages were greater than two standard deviations from the mean.

The isoproterenol- (ISO-) dependent shift in voltage dependence (ΔV_1/2_-ISO) was determined from paired recordings in individual cells before and after wash-on of 1 μM ISO in the absence or presence of PDE inhibitors. Current elicited by 1 s test pulses to −120 mV every 5–10 s was monitored during the ISO wash-on. The second GV protocol was begun when the increased current due to the ISO-dependent shift in V_1/2_ reached steady state (within 1–2 min). PDE inhibitors were present in the extracellular solution as follows: total PDE inhibition with 100 μM 3-isobutyl-1-methylxanthine (IBMX; Tocris Bioscience, Bristol, UK), PDE4 inhibition with 10 μM rolipram (roli; Tocris Bioscience) or PDE3 inhibition with 10 or 50 µM milrinone (milr; Tocris Bioscience). The PKA inhibitory peptide 6-22 amide (PKI; Tocris Bioscience) was added to the intracellular (patch pipette) solution as noted at a final concentration of 10 μM. The adenylyl cyclase inhibitor MDL-12,330A (MDL) was applied at a final concentration of 10 μM in the bath solution.

### 2.4. Statistical Analysis 

Data are presented as mean ± SEM. Statistical significance was evaluated by paired or unpaired two-tailed *t* tests or one-way ANOVAs with post-hoc tests as indicated. A *p* value of <0.05 was considered to be statistically significant.

## 3. Results

### 3.1. Phosphodiesterases Restrict Access of cAMP to HCN Channels at Rest

**Figure 1:**
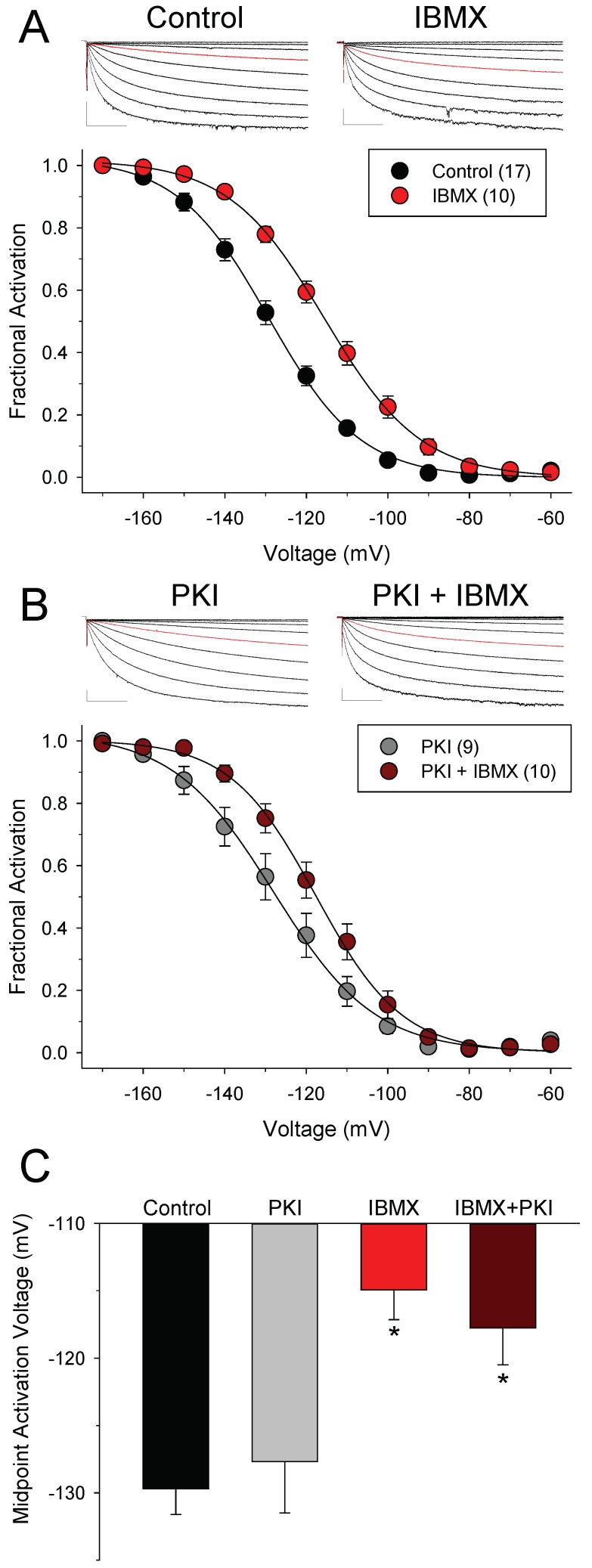
General PDE inhibition activates I_f_ in SAMs at rest via a PKA-independent mechanism. (**A,B**) Average (±SEM) normalized conductance-voltage plots for I_f_ in control (*black*), IBMX (100 µM in extracellular solution; *red*), PKI (10 µM in patch pipette; *grey*), or IBMX plus PKI (*dark red*). Numbers in parentheses in the legends indicate the number of cells in each dataset. Insets show representative I_f_ current families. Red traces show currents elicited by voltage steps to −120 mV to illustrate shifts in voltage dependence. Scale bars, 200 pA, 500 ms; and (**C**) average (±SEM) V_1/2_ for I_f_ for the indicated conditions. Asterisks indicate *p* < 0.05 versus control; one-way ANOVA with Holm-Sidak post-test.

To evaluate the overall effects of PDEs on I_f_ in mouse SAMs, we applied the general, non-subtype-selective PDE inhibitor, 3-isobutyl-1-methylxanthine (IBMX; 100 µM in the extracellular solution) to acutely isolated SAMs in whole-cell voltage-clamp experiments. We found that IBMX shifted the midpoint activation voltage (V_1/2_) of I_f_ by ~15 mV to more depolarized potentials (Figure 1A,C; Table 1), consistent with an increase in cAMP concentration in the vicinity of the HCN channels. Thus PDEs limit access of basal cAMP to HCN channels at rest.

Since we previously found that PKA activity is required for the cAMP-dependent activation of I_f_ by βARs in SAMs [11], we next asked whether PKA is also required for the cAMP liberated by IBMX to activate I_f_. To this end, we evaluated the effects of IBMX on I_f_ in SAMs in which PKA was inhibited by the pseudosubstrate inhibitory peptide, PKI 6-22 amide (PKI; 10 µM in the patch pipette). We found that IBMX had essentially identical effects in the presence or absence of PKI; the V_1/2_ of I_f_ did not differ in IBMX versus IBMX plus PKI (Figure 1C). Thus, in contrast to the cAMP generated upon βAR stimulation, the basal cAMP released upon PDE inhibition can activate HCN channels in SAMs independent of PKA activity.

### 3.2. Different Effects of PDE4 and PDE3 on I_f_ under Basal Conditions

**Figure 2:**
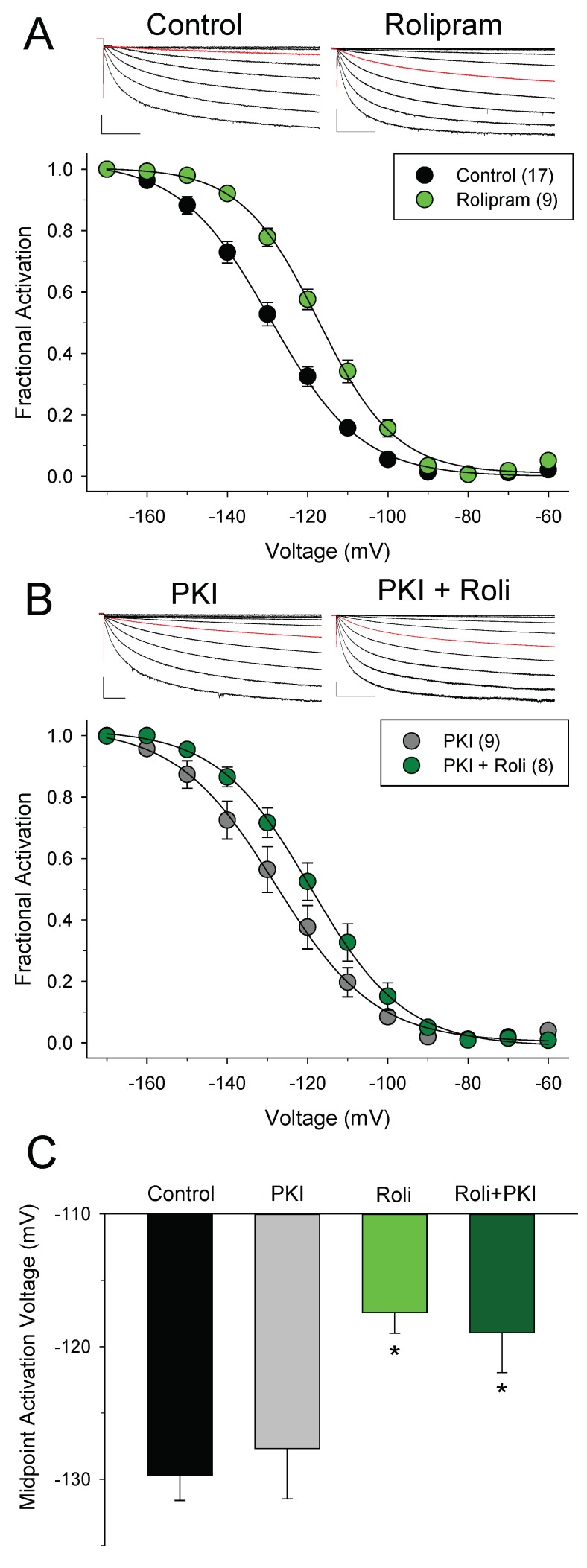
PDE4 inhibition activates I_f_ in SAMs at rest via a PKA-independent mechanism. (**A,B**) Average (±SEM) normalized conductance-voltage plots for I_f_ in control (*black*), rolipram (10 µM in the extracellular solution; *green*), PKI (10 µM in the patch pipette; *grey*), or PKI plus rolipram (*dark green*). Numbers in parentheses in the legends indicate the number of cells in each dataset. *Insets* show representative I_f_ current families. Red traces elicited by voltage steps to −120 mV illustrate shifts in the voltage dependence. Scale bars, 100 pA, 500 ms. for control, 500 pA, 500 ms for PKI; and (**C**) shows the average (±SEM) V_1/2_ for I_f_ for indicated conditions. *Asterisks* indicate *p* < 0.05 versus control; one-way ANOVA with Holm-Sidak post-test.

PDE4 activity is thought to regulate sinoatrial node pacemaker activity in a number of species, based on experiments using rolipram, a PDE4 family inhibitor [33,34,44,45]. To determine the role of PDE4 in basal regulation of I_f_ in mouse SAMs, we evaluated the effects of rolipram (10 µM in the bath solution) on the V_1/2_ of I_f_. We found that rolipram caused a significant depolarizing shift in V_1/2_ which was indistinguishable from the shift produced by IBMX (Figure 2A,C; Table 1). As in the case of IBMX, the rolipram-liberated cAMP did not require PKA activity in order to activate HCN channels, since the V_1/2_ values did not differ in rolipram alone compared to rolipram plus PKI (Figure 2C; Table 1).

**Figure 3:**
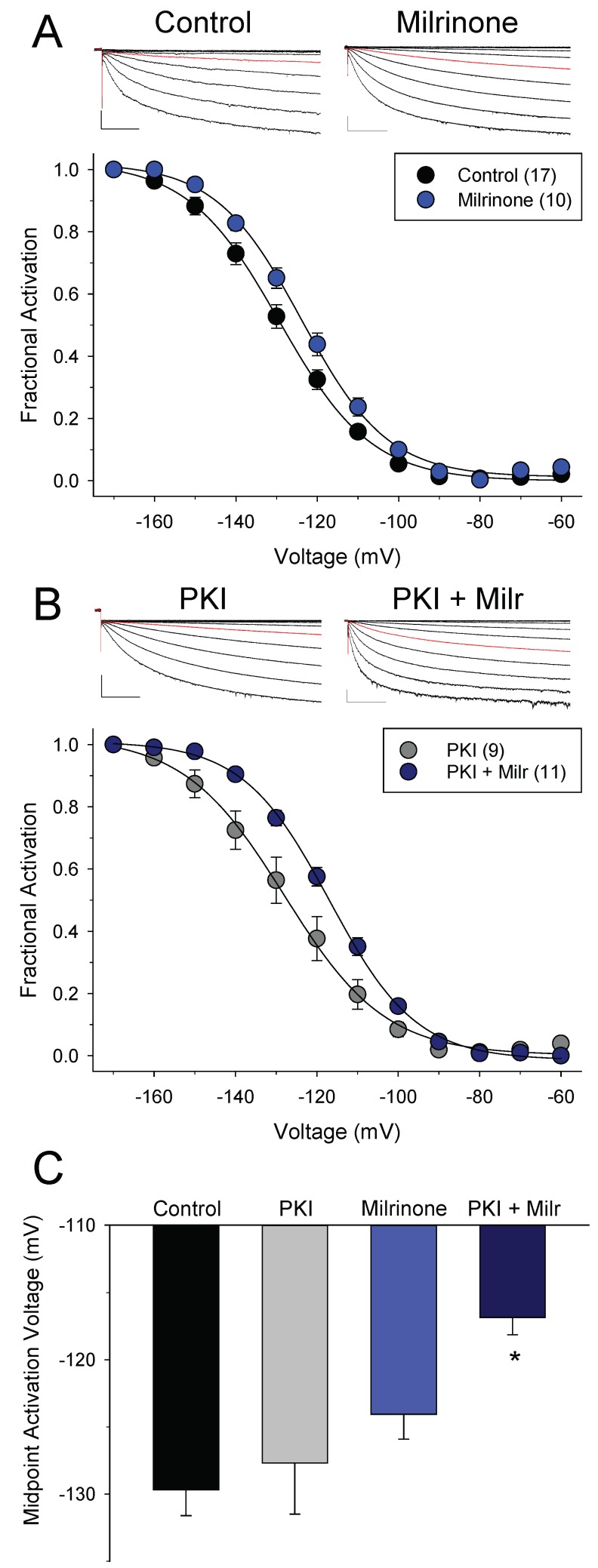
Effects of PDE3 inhibition on I_f_ at rest. (**A,B**) Average (±SEM) normalized conductance-voltage plots for I_f_ in control (black), milrinone (50 µM in the extracellular solution; *blue*), PKI (10 µM in the patch pipette; *grey*), or milrinone plus PKI (*dark blue*). Numbers in parentheses in the legends indicate the number of cells in each dataset. Insets show representative I_f_ current families. Red traces elicited by voltage steps to −120 mV illustrate shifts in the voltage dependence. Scale bars, 100 pA, 500 ms for control, 500 pA, 500 ms for PKI; and (**C**) shows the average (±SEM) V_1/2_ for I_f_ for indicated conditions. Asterisk indicates *p* < 0.05 versus control; one-way ANOVA with Holm-Sidak post-test.

The PDE3 family is also thought to affect sinoatrial node function; inhibition of PDE3 increases basal pacemaker activity in isolated SAMs and atrial preparations from mice [33,34]. In contrast to IBMX or rolipram, we found that the PDE3 family inhibitor, milrinone (10 or 50 µM) produced only a modest, statistically insignificant, depolarizing shift in the V_1/2_ of I_f_ in SAMs when applied by itself (Figure 3A,C; Table 1). However, when milrinone was applied in the presence of PKI, it produced a significant depolarizing shift in V_1/2_ of I_f_, which was similar in magnitude to the shifts produced by IBMX or rolipram (Figure 3; Table 1).

### 3.3. PDEs Establish the Requirement for PKA Activity in Signaling between βARs and HCN Channels in SAMs

**Figure 4:**
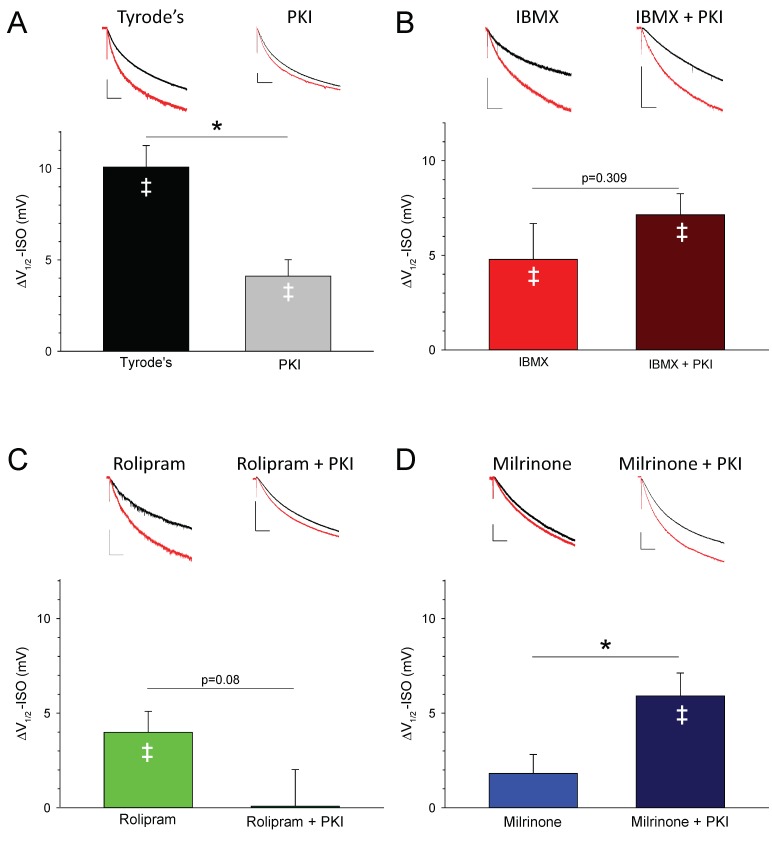
Effects of PDE inhibition on the ability of ISO to shift the voltage-dependence of I_f_. The shift in V_1/2_ of I_f_ in response to ISO (ΔV_1/2_-ISO) was determined for individual cells by measuring V_1/2_ values before and after wash-on of ISO in (**A**) control Tyrode’s extracellular solution (*black*) or Tyrode’s solution with PKI (10 µM) in the patch pipette (*grey*); (**B**) IBMX (100 µM in the extracellular solution; *red*) or IBMX with PKI in the pipette (*dark red*); (**C**) rolipram (10 µM in the extracellular solution; *light green*) or rolipram with PKI in the pipette (*dark green*); and (**D**) milrinone (50 µM; *blue*) or milrinone with PKI in the pipette (*dark blue*). Dashed lines indicate the ΔV_1/2_-ISO shift in control conditions for comparison. Asterisks indicate *p* < 0.05 compared to the corresponding condition without PKI; *t*-tests. Double daggers indicate a significant shift in response to ISO (*p* < 0.05 versus a hypothetical shift of 0 mV; one-sample *t*-tests). Insets represent the hyperpolarization-activated currents elicited by single voltage steps to near the midpoint activation voltage for each condition from individual cells before (black) and after (red) wash-on of ISO in the absence (left) or presence (right) of PKI. Scale bars: Tyrode’s, 200 pA; PKI, 200 pA; IBMX, 50 pA; IMBX + PKI, 100 pA; rolipram, 100 pA; rolipram + PKI, 200 pA; milrinone, 100 pA; milrinone + PKI, 200 pA. All time scale bars, 500 ms.

To evaluate the role of PDEs in establishing the requirement for PKA in βAR-to-HCN channel signaling in SAMs, we assayed the shift in V_1/2_ in individual cells in response to the wash-on of the βAR agonist, isoproterenol (ΔV_1/2_-ISO). ΔV_1/2_-ISO shifts were determined in the absence or presence of PDE inhibitors and PKI. In control Tyrode’s solution, the wash-on of ISO produced a ~10 mV depolarizing shift in the V_1/2_ of I_f_ (Figure 4A). In the presence of PKI in the patch pipette (but the absence of any PDE inhibitors), ISO still produced a significant shift in V_1/2_ in paired recordings from individual cells, but the magnitude of this shift was significantly reduced compared to control (Figure 4A; Table 1). In cells treated with IBMX, ISO produced a significant ~5 mV depolarizing shift in V_1/2_, in addition to the ~15 mV shift already caused by IBMX (Figure 4B; Table 1). We attribute the reduced ΔV_1/2_-ISO in IBMX versus control (Figure 4B versus Figure 4A) to a ceiling effect, because the absolute value of the V_1/2_ in IBMX plus ISO (approximately −110 mV) did not differ from that produced by a saturating concentration of cAMP introduced via the patch pipette (approximately −112 mV; Table 1). Whereas PKI significantly decreased the ΔV_1/2_-ISO under control conditions (Figure 4A), it did not reduce ΔV_1/2_-ISO when PDEs were inhibited by IBMX (Figure 4B). Thus, PDEs contribute to the formation of the PKA-dependent signaling pathway between βARs and HCN channels in SAMs, apparently by restricting the ability of βAR-stimulated cAMP to access the channels.

The contributions of the PDE4 and PDE3 families to the PKA-dependent βAR-to-HCN channel signaling pathway were evaluated using rolipram or milrinone. In cells treated with rolipram, ISO produced a significant depolarizing shift in the V_1/2_ of I_f_ (Figure 4C; Table 1). As in the case of IBMX, this ISO-dependent shift was reduced in magnitude compared to the shift in control conditions, and again, the reduction is attributed to a ceiling effect because the V_1/2_ for rolipram plus ISO did not differ from that of saturating cAMP (Table 1). However, in contrast to the situation of general PDE block with IBMX, block of PDE4s with rolipram did not relieve the requirement for PKA in βAR-to-HCN channel signaling in SAMs. In fact, PKI completely abolished the ability of ISO to shift the V_1/2_ in the presence of rolipram (*p* = 0.967 versus a hypothetical mean shift of 0; Figure 4C; Table 1). Thus, PDE4 does not appear to contribute to the PKA dependent βAR-to-HCN signaling pathway.

While the ability of ISO to shift the V_1/2_ was nearly eliminated by the PDE3 inhibitor milrinone when it was applied alone (*p* = 0.104 versus a hypothetical mean shift of 0; Figure 4D; Table 1), simultaneous inhibition of both PDE3 and PKA, with milrinone plus PKI, restored the ability of ISO to shift in V_1/2_ of I_f_ (Figure 4D; Table 1) consistent with a role for PDE3 in the formation of the PKA dependent βAR-to-HCN signaling pathway and, again, indicative of an interaction between PKA and PDE3 in the regulation of I_f_ in SAMs.

## 4. Discussion

In this study we examined the role of phosphodiesterases in cAMP-dependent regulation of I_f_ in acutely-isolated sinoatrial node myocytes from mice. We found that the PDE3 and PDE4 isoforms contribute to formation of at least two functional cAMP signaling domains that control I_f_ in SAMs. The PDE4 family restricts access of cAMP to HCN channels under basal conditions, but does not appear to play a role in the formation of the PKA-dependent βAR-to-HCN channel signaling pathway. Meanwhile, the PDE3 family interacts functionally with PKA to regulate I_f_ at rest and contributes to the formation of the PKA-dependent pathway between βARs and HCN channels in SAMs.

Our interpretation of the results assumes that the pharmacological agents we used are relatively selective and that the degree of block achieved is fairly complete. While we cannot exclude the possibility of some off-target block, our results using PKI, IBMX, rolipram, and milrinone cannot be explained by isoform cross-reactivity amongst the blockers. For example, although high concentrations of the PDE3 blocker milrinone (>10 µM) can also inhibit PDE4, lower concentrations are thought to be specific for PDE3 [33,35,46]. We tested the effects of 10 µM milrinone on I_f_, and observed a minimal response (Table 1). To ensure that we had reached a maximal effective concentration in mouse SAMs, and to compare our results to other studies [45], we also evaluated the effects of 50 µM milrinone. We found no difference in the response of I_f_ to 10 or 50 µM milrinone (Table 1 [11]). Moreover, the effects of milrinone were qualitatively different from the effects of either the general PDE inhibitor, IBMX, or the PDE4 inhibitor, rolipram. Thus, we conclude that (1) PDE3, alone, has minimal effects on I_f_ (although it interacts functionally with PKA, see below), and (2) the effects of milrinone on I_f_ in our experiments did not reflect appreciable block of PDE4.

A key finding of our study is that the shifts in the basal V_1/2_ of I_f_ produced by IBMX or rolipram were similar in the presence and absence of the PKA inhibitory peptide, PKI. The shifts produced by PDE inhibition alone (without PKA inhibition) could reflect combined effects of direct cAMP binding and PKA phosphorylation, whereas those in the presence of PKI presumably result from a direct effect of cAMP alone. We interpret the similar effects in the presence and absence of PKI as an indication that the cAMP liberated upon total PDE inhibition or PDE4 inhibition activates I_f_ without a requirement for PKA activity. However, this interpretation assumes that PKI blocks a substantial fraction of the PKA activity near HCN channels in SAMs. We feel that this assumption is justified based on our finding that PKI significantly reduced the shift in V_1/2_ in response to ISO under the same conditions (Figure 4A; Table 1; [11]). A more direct assessment of PKA activity (e.g., PKA assays in sinoatrial node homogenates) would be difficult to interpret because data from tissue extracts is a poor proxy for PKA activity within temporally- and spatially-restricted cAMP signaling domains in SAMs.

The results of the present study extend our previous observations that, although PKA activity is required for βAR signaling to HCN channels in SAMs [11], it is still possible for cAMP to activate I_f_ in SAMs even in the absence of PKA activity [19]. Taken together, the previous and new data suggest a working model in which members of the PDE4 family form a functional “barrier” that isolates HCN channels from the high basal cAMP in SAMs. Disruption of this barrier with either rolipram or IBMX permits cAMP to access HCN channels, where it activates them via a PKA-independent mechanism (presumably via direct binding to the cyclic nucleotide binding domain of the channels). In our model, PDE3 family members are proposed to form a distinct functional barrier that prevents the cAMP generated upon βAR stimulation from reaching HCN channels directly, thereby constraining βAR signaling to HCN channels to a PKA-dependent pathway. Additional work will be required to determine whether these barriers represent distinct cAMP compartments in SAMs and whether the PKA-dependent activation of I_f_ by βAR stimulation results from phosphorylation of HCN channels by PKA, or if it occurs as a result of an indirect mechanism, such as control of cAMP production—e.g., by Ca^2+^-activated adenylyl cyclases [47]—with the resulting cAMP then potentiating I_f_ by binding directly to HCN channels.

Our observations of differing effects when PDE3 and PKA were inhibited together, instead of individually, indicates a complex functional interaction between PDE3 and PKA in the regulation of I_f_ in SAMs. The data preclude a simple model in which PDE3 simply acts to restrict the cAMP source that controls PKA regulation of I_f_. Instead, there must be co-regulation between PDE3 and PKA. Possible nodes of cross-talk between PDE3 and PKA include the activation of PDE3 by PKA and the inhibition of PDE3 hydrolysis of cAMP by cGMP [35,36]. In the first scenario, inhibition of PKA with PKI would also inhibit PDE3, thereby increasing cAMP, and allowing it to act by binding directly to HCN channels. Consistent with this notion, we found that milrinone, alone, had no significant effect on the V_1/2_ of I_f_, but produced a significant depolarizing shift when it was applied in the presence of PKI (Figure 3, Table 1). Meanwhile, a role for cGMP is suggested by the observation of high levels of soluble guanylyl cyclase and cGMP in the sinoatrial node [48]. Indeed, cGMP-mediated inhibition of PDE3 has been suggested to increase cAMP concentration and accelerate AP firing rate in mouse sinoatrial nodes [49]. cGMP-mediated inhibition of PDE3 has also been shown to modulate cAMP levels in subcellular compartments involved in βAR signaling in ventricular myocytes [50].

Our data complement results of previous studies in which PDEs have been shown to regulate pacemaker activity of the sinoatrial node. Our observations of depolarizing shifts in the voltage dependence of I_f_ in response to PDE inhibition are in agreement with the notions that the resting cAMP concentration is relatively high in SAMs and that it is limited by constitutive PDE activity [5,33,45,47]. The role of PDE4 in limiting the basal cAMP concentration in the vicinity of HCN channels in our study is in agreement with the observations of Hua et al. [33], who found that inhibition of PDE4 with rolipram (10 µM) increased AP firing rate and I_Ca,L_ in isolated mouse SAMs to a greater extent than did PDE3 inhibition with milrinone (10 µM). Our data suggest that I_f_ works along with I_Ca,L_ to mediate the increase in AP firing rate in response to PDE4 inhibition. Galindo-Tovar and Kaumann [34] used rolipram (1 µM) along with the PDE3 inhibitor cilostamide (300 nM) to show that both PDE3 and PDE4 contribute to control of basal firing rate in isolated mouse right atria via actions on a cAMP compartment that is distinct from that mediated by βARs. These data are in good agreement with our observations of multiple functional cAMP signaling domains formed by PDEs in SAMs and of PDE4-dependent regulation of I_f_ under basal conditions. However, they also suggest that additional PDE3-dependent mechanisms may also contribute to pacemaker activity in the mouse sinoatrial node. Interestingly, βAR-induced tachycardia across many species is resistant to PDE inhibition, suggesting that cAMP signaling between βARs and relevant effectors for the fight-or-flight increase in heart rate is not controlled by PDEs [51,52]. Hence, βAR regulation of I_f_ appears to serve primarily as a frequency adaptation mechanism rather than a primary driver of the sympathetic heart rate response. Vinogradova et al. [45] used milrinone (50 µM) to suggest that PDE3 may be the dominant isoform controlling basal firing rate in rabbit SAMs. However, we did not observe a difference between 10 µM and 50 µM milrinone in mouse SAMs, and saw effects of milrinone only in combination with PKA inhibition. Taken together, these data suggest that there may be species-dependent differences in the roles of different PDE isoforms in regulation of sinoatrial node activity.

## 5. Conclusions

In summary, our results indicate that the PDE3 and PDE4 isoforms create functionally-distinct cAMP signaling domains in mouse SAMs that regulate I_f_ at rest and in response to βAR stimulation. Specifically, the PDE4 family restricts access of cAMP to HCN4 channels under basal conditions, and the PDE3 family contributes to the formation of a preferred PKA-dependent pathway between βARs and HCN channels. Given that I_f_ is a critical factor in sinoatrial node pacemaker activity, it is likely that these mechanisms contribute to the regulation of heart rate.

## Figures and Tables

**Table 1:** Midpoint activation voltages for I_f_ in mouse sinoatrial myocytes. V_1/2_ values for I_f_ were determined in the absence or presence of PDE inhibitors (IBMX 100 µM, rolipram 10 µM, milrinone 10 µM, or 50 µM as indicated) in the extracellular solution with and without the PKA inhibitory peptide, PKI (10 µM) in the pipette solution. For each cell, V_1/2_ values were determined before or after wash-on of 1 µM isoproterenol in the extracellular solution; ΔV_1/2(ISO)_ is the average ISO-induced shift in V_1/2_. Data for cAMP (1 mM in the patch pipette) and MDL-12,330A (10 µM in the extracellular solution) are provided for comparison from [13].

Treatment	V_1/2_ before ISO (mV)	V_1/2_ after ISO (mV)	ΔV_1/2_-ISO (mV)	n	*p* Value Control vs. ISO (Paired *t*-Test)
CONTROL	−129.7 ± 1.9	−119.6 ± 1.5	10.1 ± 1.2	17	0.000000237
+PKI	−127.7 ± 3.8	−123.6 ± 3.5	4.1 ± 0.7	9	0.00188
IBMX	−114.9 ± 2.2	−110.1 ± 2.8	4.8 ± 1.9	10	0.0328
+PKI	−117.7 ± 2.7	−110.6 ± 2.7	7.1 ± 1.1	10	0.000128
ROLIPRAM	−117.4 ± 1.6	−113.4 ± 2.2	4.0 ± 1.1	9	0.00712
+PKI	−118.9 ± 3.0	−118.8 ± 2.8	0.1 ± 1.9	7	0.967
MILRINONE (10 μM)	−124.0 ± 4.3	−121.1 ± 4.7	2.9 ± 1.7	8	0.123
+PKI	−119.3 ± 4.7	−112.5 ± 4.8	6.8 ± 2.4	10	0.0206
MILRINONE (50 μM)	−124.1 ± 1.9	−122.2 ± 2.0	1.8 ± 1.0	10	0.104
+PKI	−116.8 ± 1.3	−110.9 ± 1.8	5.9 ± 1.2	11	0.000648
MDL-12,330A	−131.1 ± 1.9	−132.0 ± 2.2	−0.9 ± 1.1	7	0.469
1 mM cAMP	−112.0 ± 1.6	-	-	10	-
3 mM cAMP	−114.1 ± 1.9	-	-	7	-

## Abbreviations

AP: action potential
βAR: adrenergic receptor
cAMP: 3’,5’-cyclic adenosine monophosphate
HCN channel: hyperpolarization-activated, cyclic nucleotide-sensitive ion channel
IBMX: 3-isobutyl-1-methylxanthine
I_f_: funny current
ISO: isoproterenol
milr: milrinone
PDE: phosphodiesterase
PKA: cAMP-dependent protein kinase
PKI: PKA inhibitory peptide
roli: rolipram
SAM: sinoatrial node myocyte
V_1/2_: midpoint activation voltage
